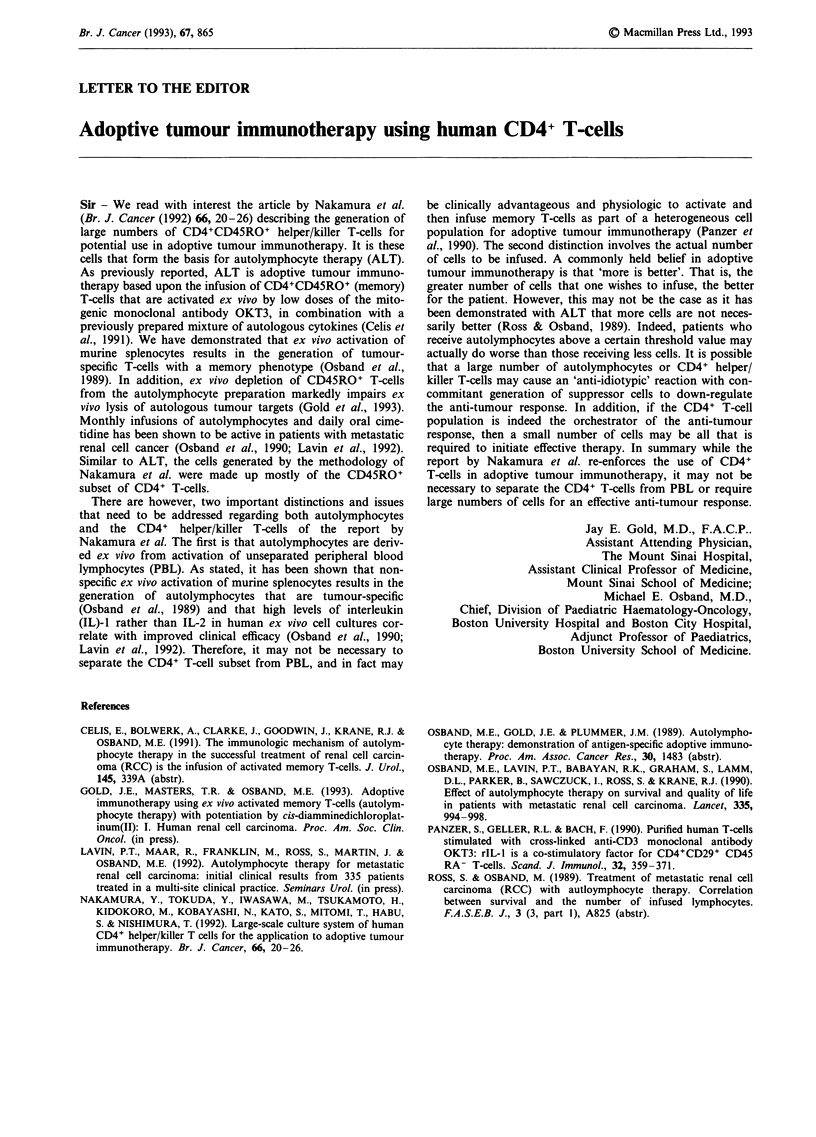# Adoptive tumour immunotherapy using human CD4+ T-cells.

**DOI:** 10.1038/bjc.1993.159

**Published:** 1993-04

**Authors:** J. E. Gold, M. E. Osband


 © Macmillan Press Ltd., 1993

### LETTER TO THE EDITOR

### Adoptive tumour immunotherapy using human CD4$^+$ T-cells

**Sir** – We read with interest the article by Nakamura *et al.* (*Br. J. Cancer* (1992) **66**, 20–26) describing the generation of large numbers of CD4$^+$CD45RO$^+$ helper/killer T-cells for potential use in adoptive tumour immunotherapy. It is these cells that form the basis for autolymphocyte therapy (ALT). As previously reported, ALT is adoptive tumour immunotherapy based upon the infusion of CD4$^+$CD45RO$^+$ (memory) T-cells that are activated *ex vivo* by low doses of the mitogenic monoclonal antibody OKT3, in combination with a previously prepared mixture of autologous cytokines (Celis *et al.*, 1991). We have demonstrated that *ex vivo* activation of murine splenocytes results in the generation of tumour-specific T-cells with a memory phenotype (Osband *et al.*, 1989). In addition, *ex vivo* depletion of CD45RO$^+$ T-cells from the autolymphocyte preparation markedly impairs *ex vivo* lysis of autologous tumour targets (Gold *et al.*, 1993). Monthly infusions of autolymphocytes and daily oral cimetidine has been shown to be active in patients with metastatic renal cell cancer (Osband *et al.*, 1990; Lavin *et al.*, 1992). Similar to ALT, the cells generated by the methodology of Nakamura *et al.* were made up mostly of the CD45RO$^+$ subset of CD4$^+$ T-cells.

There are however, two important distinctions and issues that need to be addressed regarding both autolymphocytes and the CD4$^+$ helper/killer T-cells of the report by Nakamura *et al.* The first is that autolymphocytes are derived *ex vivo* from activation of unseparated peripheral blood lymphocytes (PBL). As stated, it has been shown that non-specific *ex vivo* activation of murine splenocytes results in the generation of autolymphocytes that are tumour-specific (Osband *et al.*, 1989) and that high levels of interleukin (IL)-1 rather than IL-2 in human *ex vivo* cell cultures correlate with improved clinical efficacy (Osband *et al.*, 1990; Lavin *et al.*, 1992). Therefore, it may not be necessary to separate the CD4$^+$ T-cell subset from PBL, and in fact may be clinically advantageous and physiologic to activate and then infuse memory T-cells as part of a heterogeneous cell population for adoptive tumour immunotherapy (Panzer *et al.*, 1990). The second distinction involves the actual number of cells to be infused. A commonly held belief in adoptive tumour immunotherapy is that 'more is better'. That is, the greater number of cells that one wishes to infuse, the better for the patient. However, this may not be the case as it has been demonstrated with ALT that more cells are not necessarily better (Ross & Osband, 1989). Indeed, patients who receive autolymphocytes above a certain threshold value may actually do worse than those receiving less cells. It is possible that a large number of autolymphocytes or CD4$^+$ helper/killer T-cells may cause an 'anti-idiotypic' reaction with concommitant generation of suppressor cells to down-regulate the anti-tumour response. In addition, if the CD4$^+$ T-cell population is indeed the orchestrator of the anti-tumour response, then a small number of cells may be all that is required to initiate effective therapy. In summary while the report by Nakamura *et al.* re-enforces the use of CD4$^+$ T-cells in adoptive tumour immunotherapy, it may not be necessary to separate the CD4$^+$ T-cells from PBL or require large numbers of cells for an effective anti-tumour response.

Jay E. Gold, M.D., F.A.C.P..
Assistant Attending Physician,
The Mount Sinai Hospital,
Assistant Clinical Professor of Medicine,
Mount Sinai School of Medicine;
Michael E. Osband, M.D.,
Chief, Division of Paediatric Haematology-Oncology,
Boston University Hospital and Boston City Hospital,
Adjunct Professor of Paediatrics,
Boston University School of Medicine.

### References

CELIS, E., BOLWERK, A., CLARKE, J., GOODWIN, J., KRANE, R.J. & OSBAND, M.E. (1991). The immunologic mechanism of autolymphocyte therapy in the successful treatment of renal cell carcinoma (RCC) is the infusion of activated memory T-cells. *J. Urol.*, **145**, 339A (abstr).

GOLD, J.E., MASTERS, T.R. & OSBAND, M.E. (1993). Adoptive immunotherapy using *ex vivo* activated memory T-cells (autolymphocyte therapy) with potentiation by *cis*-diamminedichloroplatinum(II): I. Human renal cell carcinoma. *Proc. Am. Soc. Clin. Oncol.* (in press).

LAVIN, P.T., MAAR, R., FRANKLIN, M., ROSS, S., MARTIN, J. & OSBAND, M.E. (1992). Autolymphocyte therapy for metastatic renal cell carcinoma: initial clinical results from 335 patients treated in a multi-site clinical practice. *Seminars Urol.* (in press).

NAKAMURA, Y., TOKUDA, Y., IWASAWA, M., TSUKAMOTO, H., KIDOKORO, M., KOBAYASHI, N., KATO, S., MITOMI, T., HABU, S. & NISHIMURA, T. (1992). Large-scale culture system of human CD4$^+$ helper/killer T cells for the application to adoptive tumour immunotherapy. *Br. J. Cancer*, **66**, 20–26.

OSBAND, M.E., GOLD, J.E. & PLUMMER, J.M. (1989). Autolymphocyte therapy: demonstration of antigen-specific adoptive immunotherapy. *Proc. Am. Assoc. Cancer Res.*, **30**, 1483 (abstr).

OSBAND, M.E., LAVIN, P.T., BABAYAN, R.K., GRAHAM, S., LAMM, D.L., PARKER, B., SAWCZUCK, I., ROSS, S. & KRANE, R.J. (1990). Effect of autolymphocyte therapy on survival and quality of life in patients with metastatic renal cell carcinoma. *Lancet*, **335**, 994–998.

PANZER, S., GELLER, R.L. & BACH, F. (1990). Purified human T-cells stimulated with cross-linked anti-CD3 monoclonal antibody OKT3: rIL-1 is a co-stimulatory factor for CD4$^+$CD29$^+$ CD45 RA$^-$ T-cells. *Scand. J. Immunol.*, **32**, 359–371.

ROSS, S. & OSBAND, M. (1989). Treatment of metastatic renal cell carcinoma (RCC) with autloymphocyte therapy. Correlation between survival and the number of infused lymphocytes. *F.A.S.E.B. J.*, **3** (3, part 1), A825 (abstr).